# FRIENDSHIP NETWORKS AND HEALTH AMONG BLACK AND WHITE DEMENTIA CAREGIVERS

**DOI:** 10.1093/geroni/igad104.1588

**Published:** 2023-12-21

**Authors:** Yee To Ng, Kira Birditt

**Affiliations:** Institution of Social Researach, Ann Arbor, Michigan, United States; Institute for Social Research, University of Michigan, Ann Arbor, Michigan, United States

## Abstract

Social networks and support protect against health risks associated with dementia caregiving. Yet, little is known about the unique role of friends in the dementia caregiving context, as most prior caregiving studies do not differentiate between family and friends when examining social networks and support. The experience of racism and stronger familial values among Black individuals may affect their friendship networks and the implications of friends for health. This study examines racial differences in the number of friends in close social networks, contact frequency and support from friends, and links with mental health among Black and White dementia caregivers. A total of 98 dementia caregivers (63% White Americans, 90% female, Meanage = 62.66) completed baseline interviews in which their social network and mental well-being were assessed. Findings revealed that White caregivers reported more friends in their close networks than Black caregivers. However, there were no racial differences in contact frequency with network friends, or the frequency of receiving practical and emotional help from friends in assisting patients with dementia. Furthermore, receiving emotional support from friends was linked to lower levels of anxiety and depression, and these effects were greater among White caregivers. Together, this study provides a better understanding of racial disparities in friendship networks among dementia caregivers and their implications for mental health.

